# Stigmatization in teachers towards adults with attention deficit hyperactivity disorder

**DOI:** 10.1186/2193-1801-3-26

**Published:** 2014-01-14

**Authors:** Anselm BM Fuermaier, Lara Tucha, Anna K Mueller, Janneke Koerts, Joachim Hauser, Klaus W Lange, Oliver Tucha

**Affiliations:** Department of Clinical and Developmental Neuropsychology, Faculty of Behavioural and Social Sciences, University of Groningen, Grote Kruisstraat 2/1, 9712 TS Groningen, The Netherlands; Department of Experimental Psychology, University of Regensburg, Regensburg, Germany

**Keywords:** Stigmatization, Adult ADHD, Education, Teachers, Prevention, Intervention

## Abstract

**Objectives:**

Attention deficit hyperactivity disorder (ADHD) is understood as a developmental disorder which shares common characteristics between childhood, adolescence and adulthood. However, ADHD is widely associated with misconceptions and misbeliefs which can lead to stigmatization. Teachers have an important role for the individual development as they accompany students for a long period of time. The aim of the present study was to explore stigmatizing attitudes in teachers towards adults with ADHD, thereby focusing on the developmental trajectory of the condition. Furthermore, it was aimed to identify factors contributing to prevention and intervention of stigmatization in ADHD.

**Methods:**

Stigma responses of 170 teachers and 170 comparison participants were measured and compared with a recently developed tool for the assessment of stigmatization towards adults with ADHD. Furthermore, the contribution of knowledge about ADHD and the frequency of contact with adults with ADHD to stigmatization were explored.

**Results:**

Teachers showed significantly less stigmatizing attitudes than comparison participants in various dimensions, including *Reliability and Social Functioning*, *Malingering and Misuse of Medication* and the total scale. With regard to teachers, frequency of contact with adults with ADHD was not related to stigma. However, knowledge about the disorder was negatively correlated with stigma in teachers, indicating lower expressed stigma with increasing knowledge about adult ADHD.

**Conclusions:**

Teachers demonstrated more sensitized attitudes towards stigma in adults with ADHD than comparison participants. Since the present results indicate that knowledge about ADHD increase the sensitivity towards the disorder, special education programs for the community may have the potential to reduce stigmatization towards adults with ADHD. Possibilities for intervention strategies of stigmatization in educational settings were discussed.

## Introduction

### Stigmatization and ADHD

The association of a group of people with unfavorable, negative beliefs can be described as stigmatization. In this context, it is not relevant whether the assumption underlying these associations is correct or wrong. Research on stigmatization demonstrated that not only physical deviances can set individuals apart and trigger stigmatization but also intrinsic characteristics of the individual such as behavioral deviances have also been found to provoke stigma (Angermeyer et al. 2004; Rüsch 2010). Weiner and colleagues (Weiner et al. 1988) assumed that stigma deriving from individuals’ mental or behavioral deviances is even more pronounced than stigma associated with physical impairments given the stronger association between mental illness and uncontrollability and norm-violating behavior in the general public. Attention deficit hyperactivity disorder (ADHD) has been shown to be such a condition which is strongly associated with stigmatization (Mueller et al. 2012). It has been revealed that in particular externalizing and norm-violating behaviors of persons with ADHD can lead to discrimination, isolation and social rejection (dosReis et al. 2010; Koro-Ljungberg & Bussing 2009; Pescosolido et al. 2007). Moreover, not only behavioral problems linked to ADHD may elicit stigma but also the mere label of ADHD may trigger stigmatizing reactions (Banaji & Hardin 1996; Martin et al. 2007). For example, undergraduate students ratings were less socially favorable for a young adult diagnosed with ADHD than those for a person with a medical problem (e.g. asthma) or a person with an ambiguous weakness (e.g. heightened level of perfectionism) (Canu et al. 2008).

### Stigmatization towards ADHD in the educational setting

The educational setting requires students to be attentive, calm and under self-control which may cause that symptoms of ADHD become most salient. Moreover, high demands of social adjustment and organizational competence can be difficult to accomplish for individuals with ADHD. Therefore, the educational and academic setting can be assumed to set individuals with ADHD at risk of being stigmatized. Teachers have a crucial role in this environment, given their task to teach students and to assess and evaluate students’ academic performance and social skills. Therefore, stigmatizing beliefs of teachers on ADHD can cause several adverse effects. First, the competence of stigmatized persons might get more negatively judged and evaluated by teachers. Second, the teacher’s perception of a particular person might have an impact on other students’ perception of the stigmatized person (Atkinson et al. 1997). Finally, the teacher’s expectancies of a student have been shown to adversely affect the student’s performance in terms of a self-fulfilling prophecy (Rosenthal & Jacobson, 1968). Teachers’ attitudes towards disorders such as ADHD are of particular interest regarding the crucial role of teachers for the individual education and development. Teachers can be assumed to be highly experienced and educated with regard to the clinical trajectory and daily life functioning of persons with ADHD. Previous studies on stigmatization in the educational setting showed that teachers and parents hold negative assumptions on academic skills of children with ADHD. For example, Eisenberg and Schneider (2007) analyzed data from a national survey study in the United States in which teachers and parents were asked to evaluate the academic skills of children. They found that academic skills of children with ADHD were more negatively judged compared to academic skills of typically developing children even after adjusting for both academic performance (measured in math and reading standardized assessment) and children’s externalizing problem behavior (measured in teachers’ and parents’ ratings). Moreover, the mere diagnostic label of *ADHD* led to more negative judgments of the general functioning of a child (label bias) in both teachers and education students (Ohan et al. 2011). In order to reduce stigmatizing attitudes, several factors have been considered in research, including the effects of knowledge about the disorder, special education on ADHD and personal contact with individuals with ADHD. In a study by Ohan and colleagues (Ohan et al. 2011), a special training for teachers on ADHD had effects on how teachers were biased by an ADHD diagnosis (label bias) when evaluating children with ADHD. However, the effects of a special training for teachers was not consistent. For example, teachers with more ADHD specific training were less likely to be influenced by the label ADHD when asked for the willingness to support treatment interventions. In contrast, a teacher training increased the impact of the ADHD label on negative emotional reactions held by teachers. However, personal contact with children with ADHD was not found to affect the label bias in this study. In a survey approach towards the assessment of stigmatization of persons with ADHD, teachers holding special education certification were more sensitive to feelings of stigmatization of persons with ADHD than teachers without such a certification. Teaching experience, however, was unrelated to stigma scores (Bell et al. 2011; Berger et al. 2001). Considering the high persistence of ADHD into adulthood, stigmatization of ADHD might also impact on students’ academic or personal achievements when entering higher education institutes. Accordingly, Vance and Weyandt (2008) inventoried university and college professors’ conceptions on students with ADHD. Most notably, neither teaching experience nor personal contact to college students with ADHD nor a special training on ADHD had any effect on perceptions of ADHD.

### Assessment tools to measure stigmatization in ADHD

Despite the fact that stigmatization in ADHD is widely prevalent (Canu et al. 2008; Martin et al. 2007; Mueller et al. 2012), there is a considerable lack of knowledge about the content of stigmatizing attitudes. This lack of knowledge can be explained by a shortage of currently available assessment tools for the specific assessment of stigmatization towards ADHD. A questionnaire approach for measuring teachers’ stigma on individuals with ADHD was applied by Bell and colleagues using the ADHD Stigma Questionnaire (ASQ) (Bell et al. 2011). However, because it is an adaptation of a measure originally designed for persons with human immunodeficiency virus (HIV), no items specifically aiming at stigmatizing beliefs on ADHD are included. Furthermore, the ASQ does not distinguish between children, adolescence and adults with ADHD. Due to the lack of disease specific measures of stigma in ADHD, an assessment tool specifically addressing stigmatizing beliefs towards adults with ADHD was developed recently (Fuermaier et al. 2012). This questionnaire revealed that stigma towards adults with ADHD is a multidimensional concept and six dimensions of stigmatization towards adults with ADHD were introduced.

### Aim of the present study

The present study is the first to explore stigmatization of teachers towards adults with ADHD as disease specific stigmatization related to adult ADHD was difficult to assess in the past due to a lack of measurement tools. Considering that ADHD is a developmental disorder affecting childhood, adolescence and adulthood, a differentiation between different age groups appear to be necessary in the assessment of stigmatization towards persons with ADHD. It is therefore aimed to measure teachers’ stigmatization specifically towards adults with ADHD in order to obtain beliefs on the developmental trajectory of ADHD. Teachers are of particular interest as they accompany students for a long period of time and can be assumed to be a highly experienced group with regard to ADHD. Current beliefs on the developmental trajectory of ADHD and behavioral tendencies towards persons with ADHD in the educational setting can be inferred from the present study. Thus, the first study objective is to examine stigmatizing attitudes of teachers towards adults with ADHD on a disease specific assessment tool. The hypothesis that teachers show more sensitized attitudes towards adults with ADHD than comparison participants is tested on six recently developed dimensions of stigmatization. Lower stigma scores of teachers would support the sensitivity of the questionnaire in measuring stigmatization towards adults with ADHD. The second aim of the study is to examine the significance of mediating factors to stigmatization in an explorative analysis. Potential mediating factors include self-reported frequency of contact to adults with ADHD and self-rated knowledge about ADHD.

## Methods

### Participants

A total of 170 teachers and 170 comparison participants took part in the study and completed the disease specific questionnaire on stigmatization towards adults with ADHD. All teachers were recruited from local secondary schools in Germany. The teachers’ age ranged from 24 to 64 years with a mean age of 43.0 years (SD = 12.4 years). The sample of teachers consisted of 112 (65.9%) female and 58 (34.1%) male participants. All teachers achieved a university degree and completed an applied training in school education. Mean education of teachers was 19.0 years (SD = 2.5 years). Comparison participants were recruited via public announcements, word-of-mouth and through contacts of the researchers involved in Germany. The sample’s age ranged from 22 to 65 years with a mean age of 42.7 years (SD = 13.9 years). The comparison group consisted of 100 (58.8%) female and 70 (41.2%) male participants. Corresponding to the sample of teachers, all participants in the comparison group achieved a university degree with a mean education of 18.6 years in total (SD = 3.8 years). Comparison participants had a variety of professions. However, none of the comparison participants completed a study program for teachers or completed an applied training in school education and none of the comparison participants was currently working in the educational setting in a teaching position. Teachers and comparison participants did not differ in age (t(338) = 0.185, p = .853), gender (*χ*(1) = 1.804, p = .179) and educational level (t(338) = 0.974, p = .331).

### Measures

A recently developed questionnaire for the measurement of stigmatization in adults with ADHD was applied (Fuermaier et al. 2012). The questionnaire contained 37 items (statements) which were individually rated on a 6-point-Likert-scale (-3 = strongly disagree, -2 = disagree, -1 = somewhat disagree, 1 = somewhat agree, 2 = agree, 3 = strongly agree) with higher scores indicating higher stigmatization. Prior to the 37 statements, eight inventorial questions were asked to obtain general background information and descriptive information of the respondents. Information about participants’ self-rated knowledge concerning ADHD and their familiarity with individuals with ADHD (including personal contact), was obtained at the end of the questionnaire. Respondents were asked to indicate whether they have ever heard about ADHD and to specify their self-rated knowledge about ADHD on a scale ranging from 0 (no knowledge) to 10 (expert knowledge). In addition, respondents stated whether they ever had contact with an adult with ADHD as well as the frequency of contact from 0 (never) to 5 (constantly, daily). These questions were introduced at the end of the questionnaire in order to prevent biased responses of participants to the 37 statements.

As reported in a previous study (Fuermaier et al. 2012), exploratory and confirmatory factor analysis supported good model fit of a 6-factor structure of the questionnaire. In a first sample (n_1_ = 516), exploratory factor analysis (EFA) suggested a 6-factor structure in a principal component analysis (PCA). The use of a PCA was justified by adequate sample size (KMO = .86) and by sufficient large correlations between the items indicated by Bartlett’s test of sphericity (*χ*^2^(2016) = 8258.3, p < .001). The scale demonstrated adequate internal consistency (Cronbach’s α = .91) with internal consistencies of the six subscales ranging from .61 to .87. In a second sample (n_2_ = 517), confirmatory factor analysis (CFA) confirmed the proposed 6-factor model with satisfying model fit (*χ*^2^ (614) = 1763.68; p < .01; *χ*^2^/df = 2.87; RMSEA = .06; 90%-CI for RMSEA = [.057; .064]; SRMR = .07; CFI = .93). The questionnaire yields a total stigma score and the six subscales *Reliability and Social Functioning*, *Malingering and Misuse of Medication, Ability to Take Responsibility, Norm-violating and Externalizing Behavior*, *Consequences of Diagnostic Disclosure* and *Etiology*. The six subscales and included items are presented in Table 1.

**Table 1:** **Subscales and items of the applied questionnaire on stigmatization towards adult ADHD**

**Subscale 1: Reliability and social functioning**	
1	Adults with ADHD care less about other’s problems.
2	Adults with ADHD are able to take care of a group of children in kindergarten.*
3	You cannot rely on adults with ADHD.
4	Adults with ADHD are self-focused and egoistic.
5	I would go on a date with someone with ADHD.*
6	Adults with ADHD have no problems in making friends.*
7	Adults with ADHD are less successful than adults without ADHD.
8	Adults with ADHD are able to lead a group of people.*
9	Under medication, adults with ADHD are less trustworthy.
**Subscale 2: Malingering and misuse of medication**	
1	Many adults with ADHD simulate the symptoms.
2	Adults with ADHD misuse their medication (sell it to others, take too much…)
3	ADHD is invented by drug companies to make profit.
4	Many adults with ADHD exaggerate their symptoms in order to be medicated.
5	ADHD is a childhood disorder and not seen in adults.
6	Adults with ADHD lie more often than adults without ADHD.
7	Adults with ADHD have a lower IQ than adults without ADHD.
8	Many adults pretend to have ADHD just to get access to medication.
9	Adults with ADHD are less able to give advice.
**Subscale 3: Ability to take responsibility**	
1	Adults with ADHD are bad parents and have problems with raising children.
2	I would mind if my investment advisor had ADHD.
3	I would not mind if a doctor who has ADHD treated me.*
4	If I had a business, I would not hire a person with an ADHD diagnosis.
5	I would mind if the teacher of my children had ADHD.
**Subscale 4: Norm-violating and externalizing behavior**	
1	Adults with ADHD are more often involved in traffic errors.
2	I could tell when a person around me has ADHD.
3	Adults with ADHD act without thinking.
4	Adults with ADHD have a different sense of humor than adults without ADHD.
5	Adults with ADHD cannot deal with money.
**Subscale 5: Consequences of diagnostic disclosure**	
1	People’s attitudes about ADHD make persons with ADHD feel worse about themselves.
2	Adults with ADHD are of lower social status.
3	As a rule, adults with ADHD feel that telling others that they have ADHD was a mistake.
4	Adults with ADHD have a lower self-esteem than adults without ADHD.
5	Adults with ADHD feel excluded from society.
**Subscale 6: Etiology**	
1	1 ADHD is caused by bad parenthood.
2	Extensive exposure to video games and TV shows can cause ADHD.
3	Adults with ADHD do not engage enough in sports.
4	ADHD is a consequence of childhood trauma.

* = inversed items.

### Procedure

All participants were invited to take part in the study on a voluntary basis and received no reward for participation. The time to complete the questionnaire was estimated to take around twenty minutes. Before the completion of the questionnaire, participants were informed about the aim of the study and it was emphasized that all data will be analyzed anonymously. Participants were required to read and acknowledge an information sheet prior to completion of the questionnaire. Formal written consent was not sought as submission of completed questionnaires was taken as implied consent. The study was approved by the Ethical Committee Psychology (ECP) affiliated to the University of Groningen, the Netherlands.

### Statistical analysis

Stigma scores were compared between teachers and participants of the comparison group using multivariate analysis of variance (MANOVA). Effect sizes (η^2^, Cohen’s d) were calculated for all comparisons. As described by Cohen (Cohen 1988), η^2^ is a function of the effect size index f. According to Cohen (Cohen 1988), a small effect size (f = .10) corresponds to an η^2^ = .0099, a medium effect size (f = .25) to an η^2^ = .0588 and a large effect size (f = .40) to an η^2^ = .1379. For pairwise comparisons of means, negligible effects (d < 0.20), small effects (d = 0.20), medium effects (d = 0.50) and large effects (d = 0.80) were distinguished (Cohen 1988). The overall significance level was set at α = .05 (total scale). In pairwise comparisons of stigma dimensions (six subscales) between groups, multiple test procedures lead to α-error accumulation. In order to control for the problem of multiple comparisons, the significance level α was adjusted for the analysis of stigma subscales by using Bonferroni correction. Moreover, the effects of self-rated knowledge about ADHD and frequency of contact with adults with ADHD were examined. Teachers and comparison participants were compared with regard to knowledge about ADHD and frequency of contact with adults with ADHD by calculating t-tests for independent samples and effect sizes (Cohen’s d). In addition, exploratory correlation analyses were applied between stigma scores and both knowledge about ADHD and frequency of contact with adults with ADHD. According to Cohen (Cohen 1988), small effects (r = 0.1), medium effects (r = 0.2) and large effects (r = 0.5) were defined. All statistical analyses were carried out using SPSS 20 for Windows.

## Results

### Group differences of stigmatization

**Figure 1:**
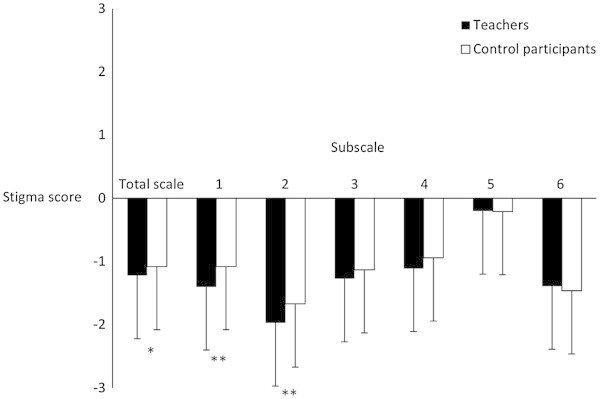
**Stigma responses of teachers and comparison participants (M ± SD).** Higher scores indicate higher stigmatization; Subscale 1: Reliability and Social Functioning; Subscale 2: Malingering and Misuse of Medication; Subscale 3: Ability to Take Responsibility; Subscale 4: Norm-violating and Externalizing Behavior; Subscale 5: Consequences of Diagnostic Disclosure; Subscale 6: Etiology; *p < .05; **p < .0083.

Overall stigma on the total scale resulted in a negative score with a mean of -1.22 (SD = 0.59) for teachers and -1.08 (SD = 0.65) for comparison participants. Teachers showed highest stigma responses on subscale 5, followed by subscale 4, subscale 3, subscale 6 and subscale 1, with the lowest observed stigma score on subscale 2. Comparison participants showed a very similar pattern of stigmatization compared to teachers, with highest stigma scores on subscale 5, followed by subscale 4, subscale 1, subscale 3, subscale 6 and subscale (Figure 1).

Multivariate analysis of variance (MANOVA) revealed a significant small difference of stigmatization between teachers and comparison participants (Wilk’s lambda = 0.894, F(7,332) = 5.604, p < .001, η^2^ = .106). Teachers expressed significant lower stigma than comparison participants on the total scale (F(1,338) = 4.35, p = .038, d = 0.23). For pairwise group comparisons of stigma scores on the six subscales, a Bonferroni adjusted significance level of α = .0083 was applied because of multiple comparisons. Teachers revealed significant lower stigma scores than comparison participants on the two subscales *Reliability and Social Functioning* (F(1,338) = 12.90, p < .001, d = 0.39) and *Malingering and Misuse of Medication* F(1,338) = 16.34, p < .001, d = 0.43). Effects were of small size. Group comparisons with regard to the other subscales revealed only non-significant differences of negligible size (*Ability to Take Responsibility*: F(1,338) = 1.17, p = .281, d = 0.12; *Norm-violating and Externalizing Behavior*: F(1,338) = 3.11, p = .079, d = 0.19; *Consequences of Diagnostic Disclosure*: F(1,338) = 0.02, p = .902, d < 0.01; *Etiology*: F(1,338) = 0.44, p = .509, d = 0.07). Stigma scores of teachers and comparison participants are presented in Figure 1.

### Effects of knowledge about ADHD and frequency of contact

The contribution of self-rated knowledge about ADHD and frequency of contact with adults with ADHD on stigma responses were examined by applying exploratory correlation analyses. Knowledge about ADHD was rated on a scale from 0 (no knowledge) to 10 (expert knowledge) and resulted in a mean score of 4.16 (SD = 1.96) for teachers and 3.77 (SD = 2.25) for comparison participants. Frequency of contact with adults with ADHD was rated on a scale from 0 (never) to 5 (constantly, daily) and resulted in mean score of 1.56 (SD = 1.41) for teachers and 1.39 (SD = 1.42) for comparison participants. Differences between teachers and comparison participants were non-significant and of negligible size for both knowledge about ADHD (t (311) = 1.443, p = .150, d = 0.18) and frequency of contact with adults with ADHD (t (306) = 1.042, p = .298; d = 0.12).

With regard to teachers, significant negative correlations were found between knowledge about ADHD and stigma scores on the total scale (r = -0.19, p = .049), on subscale 1 (r = -0.23, p = .014) and on subscale 3 (r = -0.21, p = .029). Associations were of small to medium size. Non-significant negligible to small correlations were found between self-rated knowledge and stigma responses on subscale 2 (r = -0.15, p = .126), subscale 4 (r = .02, p = .852), subscale 5 (r = -0.16, p = .088) and subscale 6 (r = -.16, p = .088). Moreover, with regard to the group of teachers, no significant correlations were obtained between frequency of contact with adults with ADHD and stigma responses (Table 2).

**Table 2:** **Correlations between knowledge about ADHD and stigma scores as well as between frequency of contact with adults with ADHD and stigma scores (teachers)**

	Correlation coefficients r (p)	
Subscale	Knowledge about ADHD	Frequency of contact with adults with ADHD
1	r = -.23 (p = .014*)	r = -.15 (p = .066)
2	r = -.15 (p = .126)	r = -.07 (p = .440)
3	r = -.21 (p = .029*)	r = -.08 (p = .364)
4	r = .02 (p = .852)	r = .12 (p = .154)
5	r = -.05 (p = .622)	r = -.10 (p =. 241)
6	r = -.16 (p = .088)	r = -.09 (p = .266)
Total scale	r = -.19 (p = .049*)	r = -.09 (p = .277)

Subscale 1: Reliability and Social Functioning; Subscale 2: Malingering and Misuse of Medication; Subscale 3: Ability to Take Responsibility; Subscale 4: Norm-violating and Externalizing Behavior; Subscale 5: Consequences of Diagnostic Disclosure; Subscale 6: Etiology; *Significant at p < .05.

With regard to comparison participants, significant correlations of medium size were obtained between knowledge about ADHD and stigma responses on subscale 1 (r = -.20, p = .017) and on subscale 4 (r = .24, p = .004). The remaining subscales of the stigma questionnaire did not correlate significantly with knowledge about ADHD (subscale 2: r = -.12, p = .145; subscale 3: r = -.10, p = .239; subscale 5: r = -.13, p = .118; subscale 6: r = -.13, p = .108 and the total scale: r = -.11, p = .185). Furthermore, the observed correlations were of small size. Significant negative correlations were found for comparison participants between frequency of contact with adults with ADHD and stigma responses on subscale 1 (r = -.23, p = .006), on subscale 2 (r = -.18, p = .028), on subscale 3 (r = -.25, p = .003), on subscale 6 (r = -.26, p = .002) and on the total scale (r = -.22, p = .008). Correlations were of small to medium size. The remaining subscales (subscale 4: r = .11, p = .187 and subscale 5: r = -.08, p = .328) did not correlate significantly with frequency of contact. The observed correlations were of negligible to small size (Table 3).

**Table 3:** **Correlations between knowledge about ADHD and stigma scores and between frequency of contact with adults with ADHD and stigma scores (comparison participants)**

	Correlation coefficients r (p)	
Subscale	Knowledge about ADHD	Frequency of contact with adults with ADHD
1	r = -.20 (p = .017*)	r = -.23 (p = .006*)
2	r = -.12 (p = .145)	r = -.18 (p = .028*)
3	r = -.10 (p = .239)	r = -.25 (p = .003*)
4	r = .24 (p = .004*)	r = .11 (p = .187)
5	r = -.13 (p = .118)	r = -.08 (p = .328)
6	r = -.13 (p = .108)	r = -.26 (p = .002*)
Total scale	r = -.11 (p = .185)	r = -.22 (p = .008*)

Subscale 1: Reliability and Social Functioning; Subscale 2: Malingering and Misuse of Medication; Subscale 3: Ability to Take Responsibility; Subscale 4: Norm-violating and Externalizing Behavior; Subscale 5: Consequences of Diagnostic Disclosure; Subscale 6: Etiology; *Significant at p < .05.

## Discussion

### Stigmatization in teachers and comparison participants

Stigmatization towards individuals with ADHD is an important topic since it can lead to discrimination, isolation and social rejection (dosReis et al. 2010; Koro-Ljungberg & Bussing 2009; Pescosolido et al. 2007). The educational setting is of particular interest because of its importance for individuals to achieve educational degrees and occupational skills in this environment. Moreover, individuals acquire certain social skills at schools and start building up their social network. Both teachers and comparison participants showed negative stigma scores on the total questionnaire and on all dimensions of stigmatization (six subscales). These scores indicate that the degree of stigmatization towards adults with ADHD is low to moderate in both teachers and comparison participants. Comparing stigma responses across dimensions, a similar pattern of attitudes and beliefs could be revealed for the two groups. Both groups presented with the most pronounced stigmatization on the dimension *Consequences of Diagnostic Disclosur*e, indicating that the mere diagnostic label is believed to cause adverse consequences for individuals with ADHD such as setting them apart from the general public. This is supported by previous research on stigmatization of children with ADHD, showing that the disclosure of the diagnostic status can adversely affect judgments of both the general public and teachers towards individuals with ADHD (Banaji & Hardin 1996; Martin et al. 2007; Ohan et al. 2011). In contrast, lowest stigma scores were obtained on the dimension *Malingering and Misuse of Medication*. In this respect, believes about persons with ADHD simulating symptoms and misusing medication were less pronounced than stigmatizing beliefs on the remaining dimensions. Hence, stigmatizing attitudes towards adults with ADHD does not particularly question the existence of ADHD as a real disorder but concentrates more on the behavioral characteristics of ADHD and associated consequences of its disclosure in public.

Comparison of stigma responses between teachers and comparison participants revealed significantly lower stigmatization of teachers on dimensions of *Reliability and Social Functioning* and *Malingering and Misuse of Medication*. The differences between teachers and comparison participants were of small size which is not surprising considering that both groups had a comparable high level of education and considering that higher levels of education protect against stigmatizing attitudes (McLeod et al. 2007; Pescosolido et al. 2008). Nonetheless, teachers expressed less pronounced concerns about the social skills of adults with ADHD. Behavioral characteristics of persons with ADHD within interpersonal relationships, such as being reliable, trustworthy or empathic were more positively evaluated by teachers than by comparison participants. This difference can be crucial as schools and other educational institutes represent an environment in which children, adolescents and adults do not only aim for academic degrees but also develop social skills which form the foundation of social networks and interpersonal relationships. Teachers can make a considerable contribution to the social development of their students by creating an appropriate atmosphere and environment and by supporting individuals. Therefore, the more positive evaluation of the social functioning of adults with ADHD expressed by teachers as found in the current study might be crucial for the social development of individuals with ADHD in the classroom. A slightly larger difference between teachers and comparison participants was found on the dimension *Malingering and Misuse of Medication*, indicating that teachers had fewer concerns about the existence of ADHD as a real disorder and expressed fewer notions about the misuse of an ADHD diagnosis and prescribed medication. Stigmatizing beliefs in public may be encouraged by findings of symptom exaggeration in about 48% of adult students who referred themselves to campus-based clinics for ADHD evaluation (Sullivan et al. 2007). Differences between teachers and comparison participants on these two dimensions (*Reliability and Social Functioning* and *Malingering and Misuse of Medication*) resulted in a significant lower total stigma score for teachers (i.e. overall less stigmatizing beliefs were held by teachers). Furthermore, these group differences support the sensitivity of the questionnaire in measuring stigma towards adults with ADHD and thereby underline the usefulness of the stigma questionnaire for future research.

### The effects of knowledge about ADHD and frequency of contact

To measure the teachers’ personal experiences with adult ADHD, participants were asked to quantify the frequency of contact with an adult suffering from ADHD on a scale ranging from 0 (never) to 5 (constantly, daily). Teachers’ personal experiences have not been assessed via their general teaching experience. This approach has been chosen as it can be assumed that most contact teachers have had with individuals with ADHD within school was related to children with ADHD. Since the present study focuses on adults with ADHD, the general teaching experience would therefore not lead to a realistic estimation of teachers’ experiences with adults with ADHD. Moreover, even though it is widely assumed that the actual number of ADHD students taught in the past is directly related to years of teaching experience, it has been emphasized that this relationship is not significant (Kos et al. 2004).

Data analysis revealed that the frequency of contact to adults with ADHD was not significantly related to stigma scores of teachers, while small to medium significant relationships were found on four dimensions and the total score for comparison participants, indicating decreased stigmatization with an increased frequency of contact. The lack of significant associations in teachers might have resulted from a rather frequent contact of teachers with children with ADHD and their parents who might partly also have suffered from the condition. Furthermore, teachers might have engaged with the topic of ADHD from an educational perspective via professional books, education programs as well as discussions with colleagues (Bell et al. 2011; Ohan et al. 2011). Special training in ADHD related topics might have resulted in a deeper understanding of ADHD and by this reduced or even prevented stigmatizing beliefs and attitudes in the present teacher sample. Additional contacts of teachers with adults with ADHD may, therefore, not have a considerable additional contribution on stigmatizing attitudes towards persons with ADHD. With regard to the general public, one may assume that more frequent contact with adults with ADHD may directly reduce stigmatizing attitudes, because the general public has less opportunities and therefore also less contacts with individuals (e.g. children) suffering from ADHD. Furthermore, with regard to teachers, knowledge about ADHD was associated with stigmatization towards adults with ADHD on dimensions of *Reliability and Social Functioning*, *Ability to Take Responsibility* and on the total stigma scale. The more teachers knew about ADHD the less they stigmatized on particular dimensions as indicated by small to medium effects. The present results on teachers’ *knowledge about ADHD* and *frequency of contact to adults with ADHD* are in line with the literature on educational approaches to the reduction of stigmatization. This literature reported positive effects of special trainings and education certifications on stigmatization of individuals with ADHD, whereas increased personal experiences with persons with ADHD have been shown to be ineffective (Bell et al. 2011; Ohan et al. 2011; Vance & Weyandt 2008). Knowledge about ADHD in the comparison group of the present study yielded mixed results which are difficult to interpret as two significant correlations were obtained in opposite directions, resulting in a non-significant relationship between knowledge about ADHD and stigma scores as expressed by the total stigma score.

## Conclusions

In conclusion, even though previous research showed that teachers frequently present with incomplete knowledge about ADHD (Bekle 2004; Kos et al. 2004; Sciutto et al. 2000), the present results nevertheless demonstrate a low level of stigmatization towards adults with ADHD and emphasize the sensitized attitudes of teachers towards adults with ADHD compared to a matched comparison group. Furthermore, the present results support the relevance of knowledge as a mediating factor of stigmatization and by this the importance of increasing special education and training programs that aim to increase the individuals’ knowledge about the disorder. Teachers could be specifically informed by offering additional training courses, providing special education programs, seminars, and workshops or by providing relevant information via flyers, brochures or special online platforms which are certified for their contents. Education programs should have an emphasis on the etiology of ADHD as it has been repeatedly advocated that information about etiology might reduce stigmatization (Burch 2004; Kendall 1998). Finally, the relevance of education programs for teachers is emphasized by studies showing that most teachers stated that they do not have opportunities to learn about ADHD or similar conditions. They further stated that they would benefit from such programs and indicated their interest and willingness for participation in this kind of programs (Bekle 2004; Kos et al. 2004).

### Limitations and future directions

The present study must be viewed in the context of some limitations. Knowledge about ADHD was assessed with a self-rated scale rather than an objective measure of knowledge. The validity of self-evaluations can of course be questioned. A response bias in teachers’ self-evaluations about ADHD is reported by Kos et al. (2004) who demonstrated that the actual knowledge about ADHD in pre-service and in-service teachers was significantly higher than their self-rated level of knowledge. Future studies should, therefore, take objective criteria such as scores on objective tests of knowledge or special certifications on ADHD into consideration. Similarly, the frequency of contact to adults with ADHD was measured on the basis of a self-rated scale. The aim of future studies could be to objectify the frequency of contact with individuals with ADHD in such a way that experiences in the professional setting (e.g. at school) can be differentiated from experiences in the private setting (e.g. family, friends and acquaintances). Furthermore, the empirical consequences and the behavioral significance of the demonstrated effects on the stigma questionnaire remain unclear. Teachers and comparison participants differed in stigmatizing beliefs towards adults with ADHD. However, it appears important to ascertain whether these effects manifest in actual discrediting behavior towards individuals with ADHD. Finally, future research evaluating stigmatization of teachers towards ADHD should clarify possible effects of teachers’ characteristics, such as teachers’ college education, teacher training, experiences in daily practice or additional qualifications (e.g. special education). It appears also important to explore possible differences between teachers of different types of schools. Teachers for children with special needs, teachers for primary school, high school or college teachers may evaluate the concept of ADHD in a distinct way which could lead to differences in stigmatizing attitudes and behavior and may require different prevention and intervention strategies.
